# Prediction of Breast Cancer Recurrence Using a Deep Convolutional Neural Network Without Region-of-Interest Labeling

**DOI:** 10.3389/fonc.2021.734015

**Published:** 2021-10-21

**Authors:** Nam Nhut Phan, Chih-Yi Hsu, Chi-Cheng Huang, Ling-Ming Tseng, Eric Y. Chuang

**Affiliations:** ^1^ Bioinformatics Program, Taiwan International Graduate Program, Institute of Information Science, Academia Sinica, Taipei, Taiwan; ^2^ Graduate Institute of Biomedical Electronics and Bioinformatics, National Taiwan University, Taipei, Taiwan; ^3^ Bioinformatics and Biostatistics Core, Centre of Genomic and Precision Medicine, National Taiwan University, Taipei, Taiwan; ^4^ Department of Pathology and Laboratory Medicine, Taipei Veterans General Hospital, Taipei, Taiwan; ^5^ School of Medicine, National Yang-Ming University, Taipei, Taiwan; ^6^ College of Nursing, National Taipei University of Nursing and Health Sciences, Taipei, Taiwan; ^7^ Comprehensive Breast Health Center, Taipei Veterans General Hospital, Taipei, Taiwan; ^8^ Institute of Epidemiology and Preventive Medicine, College of Public Health, National Taiwan University, Taipei, Taiwan; ^9^ Master Program for Biomedical Engineering, China Medical University, Taichung, Taiwan

**Keywords:** deep learning, whole slide image, pathology, 70-gene signature, transfer learning, label-free

## Abstract

**Purpose:**

The present study aimed to assign a risk score for breast cancer recurrence based on pathological whole slide images (WSIs) using a deep learning model.

**Methods:**

A total of 233 WSIs from 138 breast cancer patients were assigned either a low-risk or a high-risk score based on a 70-gene signature. These images were processed into patches of 512x512 pixels by the PyHIST tool and underwent color normalization using the Macenko method. Afterward, out of focus and pixelated patches were removed using the Laplacian algorithm. Finally, the remaining patches (n=294,562) were split into 3 parts for model training (50%), validation (7%) and testing (43%). We used 6 pretrained models for transfer learning and evaluated their performance using accuracy, precision, recall, F1 score, confusion matrix, and AUC. Additionally, to demonstrate the robustness of the final model and its generalization capacity, the testing set was used for model evaluation. Finally, the GRAD-CAM algorithm was used for model visualization.

**Results:**

Six models, namely VGG16, ResNet50, ResNet101, Inception_ResNet, EfficientB5, and Xception, achieved high performance in the validation set with an overall accuracy of 0.84, 0.85, 0.83, 0.84, 0.87, and 0.91, respectively. We selected Xception for assessment of the testing set, and this model achieved an overall accuracy of 0.87 with a patch-wise approach and 0.90 and 1.00 with a patient-wise approach for high-risk and low-risk groups, respectively.

**Conclusions:**

Our study demonstrated the feasibility and high performance of artificial intelligence models trained without region-of-interest labeling for predicting cancer recurrence based on a 70-gene signature risk score.

## Introduction

Breast cancer is one of the most common cancer types found in women worldwide (1). Although the overall survival rate of breast cancer has improved in the last decade, prognostication regarding the risk of recurrence and potential biomarkers for assisting clinical treatment decision have also been the focus of ongoing research (2). Thus, there are numerous studies reporting novel biomarkers and subtyping breast cancer according to recurrence risk (3, 4). The standard method for breast cancer classification uses immunohistochemistry (IHC) markers such as progesterone receptor (PR), human epidermal growth factor receptor II (HER2), and estrogen receptor (ER) together with Ki67 (5, 6). Other gene expression-based approaches such as PAM50, TargetPrint, MammaPrint, and BluePrint are also available options (7–9). However, these subtyping methods require analyzing mRNA expression levels using microarray platforms and clustering certain pre-selected genes for designating subtypes. Each method has its own advantages in assisting clinical treatment decisions. However, these methods are time-consuming and costly.

The MammaPrint and BluePrint tests are established assays for predicting high and low recurrence risk and subtyping breast cancer into basal, HER2, and luminal (9). In the WGS-PRIMe study, Mammaprint and BluePrint tests had high impact in assisting physicians making their treatment recommendations for early-stage luminal breast cancer patients, with over 92% adherence to the Mammaprint risk assessment for both low- and high-risk patients (10). In addition to this study, the IMPACt and MINDACT studies shown high concordance between treatment decisions and Mammaprint risk score for determining the necessity of adjuvant chemotherapy (over 88% for low-risk patients and over 78% for high-risk patients) (11–13). This evidence proves the power and impact of this genetic test in guiding clinical treatment decisions.

In recent years, numerous studies using artificial intelligence (AI) tools for various biological problems have been documented (14–17). There are two subdomains of AI, namely machine learning and deep learning, which use different approaches for feature selection during model training (18). While the machine learning approach requires domain knowledge to select significant features, deep learning is equipped with auto-feature extraction capability to learn the differences between groups for prediction and classification tasks without prior knowledge (18). The applications of machine learning and deep learning have been shown to have enormous impact in biomedical research (19, 20). Over the past few years, deep learning methods have matured and are now well-recognized in many biomedical fields of study (21, 22). The majority of these studies used biomedical images such as pathological (23), radiological (24, 25), and digital slide images (26) to train a convolutional neural network (CNN). Deep learning methods can also use other data types, such as DNA and RNA sequencing data and proteomics data, in either raw or processed format, to train a CNN (15, 27–29). Furthermore, the dream of using pathological images with deep learning to predict patient outcomes has been fulfilled recently (30). Leveraging the plethora of biological data could facilitate both the CNN training process and independent validation, which will in turn boost model performance even higher than the domain-expert level.

To continue the trend of integrating clinical and genetic data with AI, as well as assist physicians in making treatment recommendations, we tasked a deep neural network with predicting high and low risk of recurrence from pathological images of breast cancer patients. In addition, we also developed a novel deep learning pipeline using whole slide images (WSIs) as the only data source, without any input from pathologists for tumor region labeling.

## Materials and Methods

**Figure 1:**
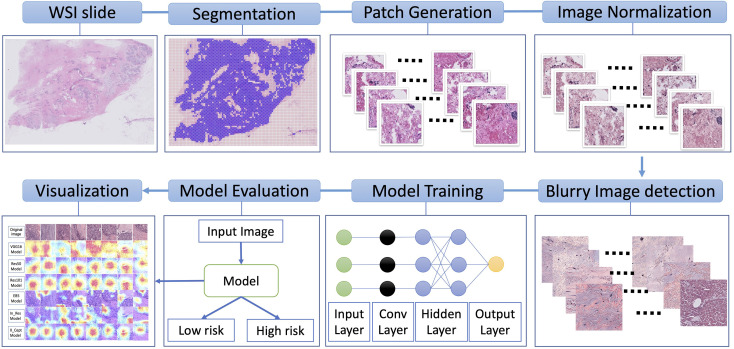
Overall schematic workflow. The whole slide images (WSI) were used to generate smaller patches of 512x512 pixels. These small patches then underwent normalization for hematoxylin and eosin staining. Next, blurry and pixelated images were removed using the Laplacian algorithm. The retained images were used for model training, validation, and testing. Finally, to illustrate how different models learn to distinguish low-risk and high-risk samples, we used gradient-weighted class activation to create a heatmap for each image.

The workflow of our study is depicted in **Figure 1**. The WSIs underwent image segmentation for patch selection using the Otsu algorithm (31). Next, selected patches of 512x512 pixels were generated. These small patches then underwent normalization for hematoxylin and eosin staining using the Macenko method (32) and a Python script from (https://github.com/schaugf/HEnorm_python) with appropriate modifications. Next, blurry and pixelated images were removed using the Laplacian algorithm. The retained images were used for model training, validation, and testing. Finally, to visualize how different models learn to distinguish samples with low-risk and high-risk 70-gene signature scores, we used gradient-weighted class activation mapping (GRAD-CAM) to create the activated heatmap for each image.

### Samples

A total of 233 WSIs from 138 breast cancer cases from Taipei Veterans General Hospital were used for model training, validation, and testing. Tumor sections from each patient were obtained and prepare for hematoxylin and eosin staining. Afterward, the stained slides were scanned with an Ultra-Fast Scanner (Philips, USA) to provide the digital slides in TIFF format. These WSIs were then used for generating patches. The same slide which performs 70 gene signature was scanned for our study. The patients’ demographics and other information such as age, nottingham grade, estrogen receptor status, progesterone receptor status, HER2 status, and TNM stage are shown in **Table 1**.

**Table 1:** The patients’ demographic information of the cohort dataset.

	Training-High Risk	Training-Low Risk	Validation-High Risk	Validation-Low Risk	Testing-High Risk	Testing-Low Risk	Total n = 138
	n = 30	n = 39	n = 6	n = 8	n = 20	n = 35	
Age, median (range)	53 (39, 86)	54 (35, 72)	63 (40, 72)	59 (35, 69)	54 (27, 76)	51 (41, 78)	53 (27, 86)
Nottingham grade							
1	3 (10%)	4 (10%)	0 (0%)	3 (37.5%)	3 (15%)	12 (34%)	25 (18%)
2	22 (73%)	34 (87%)	1 (17%)	5 (62.5%)	15 (75%)	23 (66%)	100 (73%)
3	5 (17%)	1 (3%)	5 (83%)	0 (0%)	2 (10%)	0 (0%)	13 (9%)
Estrogen receptor +	30 (100%)	39 (100%)	6 (100%)	8 (100%)	20 (100%)	35 (100%)	138 (100%)
Progesterone receptor							
+	21 (70%)	37 (95%)	4 (67%)	7 (87.5%)	18 (90%)	30 (86%)	117 (85%)
–	9 (30%)	2 (5%)	2 (33%)	1 (12.5%)	2 (10%)	5 (14%)	21 (15%)
HER2 –	30 (100%)	39 (100%)	6 (100%)	8 (100%)	20 (100%)	35 (100%)	138 (100%)
TNM Stage							
Stage I	20 (67%)	14 (36%)	5 (83%)	4 (50%)	13 (65%)	18 (51%)	74 (54%)
Stage II	10 (33%)	25 (64%)	1 (17%)	4 (50%)	7 (35%)	17 (49%)	64 (46%)

### Patch Generation

Patches from each WSI were prepared with PyHIST (33), which is a Python-based tool allowing us to select the patch at designed dimensions. We set 512x512 pixels as our patch size, and these patches were obtained at the highest magnification level (20x). Mask down-sampling and tile crossed image down-sampling were set as the defaults. The Otsu algorithm was used as the tile generation method. We set the tissue content threshold at 0.85 to select patches composed of at least 85% tissue. A total of 294,562 patches were generated from 233 WSIs, 57% of which (n=169,161) were used for model training and fine tuning. The patches were divided into a training set consisting of 160,000 patches (n= 39 (100,000 patches) for low-risk group, n = 30 (60,000 patches) for high risk group) and a validation set with 9161 patches. The other 43% of the patches (n=125,401), of which 54,122 and 71,279 came from 20 high-risk patients and 35 low-risk patients, respectively, were used for independent testing of the model performance.

### Removal of Blurry and Pixelated Images

The WSIs had blurry regions that were locally out of focus. To overcome this problem, we used the Laplacian algorithm for detecting blurry images based on variance thresholding. The kernel size of the Laplacian operator was 13x13 pixels, which was obtained by a trial-and-error approach from 3x3 pixels to 15x15 pixels. The variance threshold was set at >1e15 and <1e14. These steps were done with a custom Python script using the OpenCV library (34). After blurry and pixelated images were removed, we double-checked the whole dataset manually to make sure this threshold could remove all of these images.

### Model Training

To speed up the training process, we applied transfer learning using weights from 6 pre-trained models, namely VGG16, Res50, Res101, In_Res, X_Cept, and EB5. These models have achieved high accuracy with the ImageNet dataset (35), which is used as a common model performance benchmark. The purpose of using multiple trained models’ architecture was to take advantage of the high-performance architecture of these models as well as to compare their performance in pathological image classification.

The original patch size of 512x512 pixels was rescaled to 128x128 pixels for model input. The architecture from the pre-trained models was kept as it was; however, we used 2 fully connected layer with 1,024 neurons, and 256 neurons to reduce computational load. The second fully connected layer was connected to another hidden layer with one neuron to output the model prediction value. The thresholds of 0.3, 0.5, and 0.7 were applied for sigmoid function. Depending on the selected threshold, the model prediction, for a class with score < 0.3, <0.5 or <0.7 was high-risk group, else low-risk group.

We used adaptive moment estimation (Adam) as the optimizer with a learning rate of 1e-5, together with a decay rate of 1e-5/50 for 50 epochs at a batch size of 64. The rectifier linear unit (relu) was used as the activation function in the hidden layers, whereas sigmoid activation was used in the dense output layer.

### Model Prediction Visualization

We used gradient weighted class activation mapping (GRAD-CAM) to illustrate the model prediction visualization. The overall idea of GRAD-CAM is to use the final convolutional layer of the model to extract information on how the model made its decision for the final output class (36). After we trained our model with the WSIs data and obtained the final optimal weight file, we used this weight to obtain the GRAD-CAM visualization with the last convolutional layer in our model.

## Results

### Model Training and Validation

**Figure 2:**
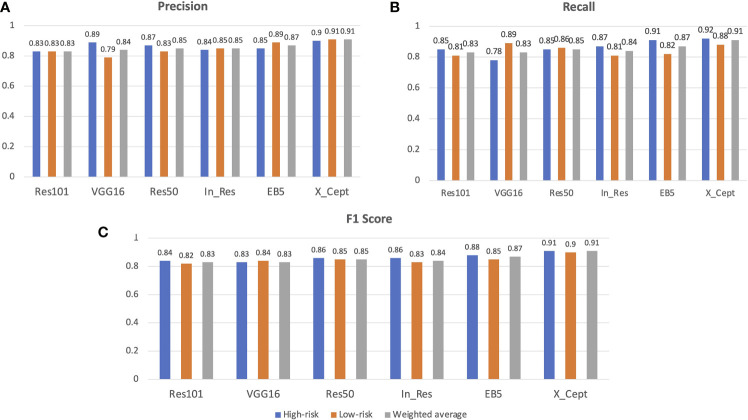
AI model performance evaluation with the validation set. Precision, recall, F1 score and confusion matrix of 6 models, namely VGG16, Res50, Res101, EB5, In_Res, and X_Cept, were used to evaluate model performance along with the accuracy metric. **(A)** Precision, **(B)** recall, and **(C)** F1 score of the 6 models in identifying the high-risk and low-risk groups. The weighted average of precision is also shown.

The results from our model training with the VGG16, Res50, Res101, EB5, In_Res, and X_Cept pretrained models achieved 83%, 85%, 83%, 87%, 85%, and 91% accuracy, respectively. These results were generated with the validation set, which proved the model was not overfit to the training and validation sets. Apart from the accuracy metric, other model evaluation metrics were also calculated, such as the weighted precision (**Figure 2A**), weighted recall (**Figure 2B**), and weighted F1 score (**Figure 2C**). The precision metric is the ratio of true positives to the sum of true positives and false positives from the model prediction, whereas recall is the ratio of true positives to the sum of true positives and false negatives from the model prediction. The F1 score is the harmonic average of precision and recall. In our study, the lowest precision, recall, and F1 score at 83% came from Res101 model and the highest at 91% was from the X_Cept model (**Figure 2**).

**Figure 3:**
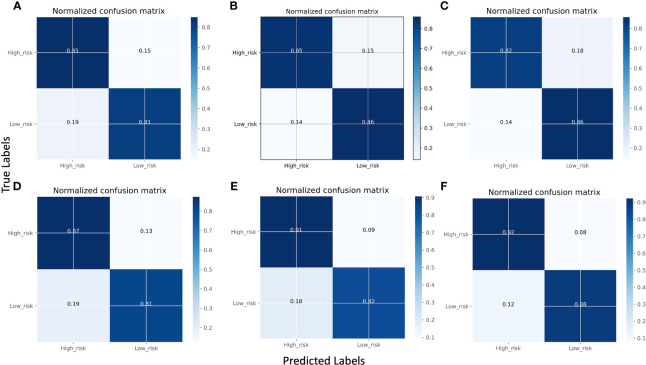
Comparison of model performance using normalized confusion matrices using the validation set. **(A)** VGG16, **(B)** Res101, **(C)** Res50, **(D)** In_Res, **(E)** EB5, and **(F)** X_Cept. The confusion matrix displays the predicted classes on the X-axis and the true classes on the Y-axis, with the color of the diagonal blocks illustrating the closeness of the match between the predicted and the true class. The darker the blue color of the diagonal line, the better the model prediction accuracy.

**Figures 3A–F** displays the normalized confusion matrix of each model. A confusion matrix shows the true labels and predicted labels of each class as well as the percentage of true positive, false positive, true negative, and false negative predictions. The darker blue color indicates a higher correct prediction of each class. The lowest-performing model was Res101 and the highest-performing model was X_Cept. For instance, Res101 had 15% false positive and 19% false negative predictions, while the X_Cept model had only 8% false positives and 12% false negatives when predicting high and low risk breast cancer patients.

**Figure 4:**
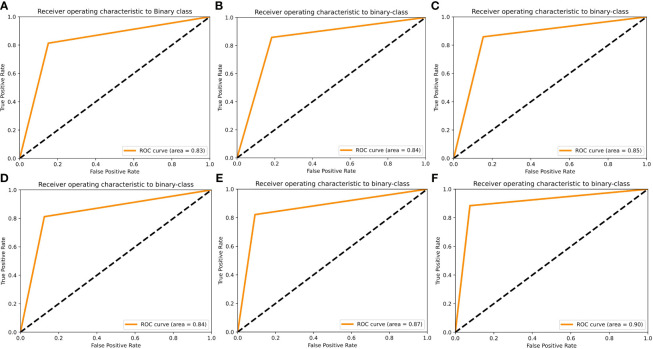
Receiver operating characteristic (ROC) curves of the 6 AI models using the validation set. ROC curves are shown for **(A)** Res101, **(B)** VGG16, **(C)** Res50, **(D)** In_Res, **(E)** EB5, and **(F)** X_Cept.

To further visualize the true positive rate and false positive rate of each model on the validation set, we also plotted the receiver operating characteristic (ROC) curve of each model (**Figures 4A–F**). The highest area under the curve (AUC) was 0.90, which belonged to the X_Cept model, whereas the lowest belonged to Res101 (AUC=0.83). The In_Res, and VGG16 models had the same AUC of 0.84, whereas Res50 and EB5 had an AUC of 0.85 and 0.87, respectively.

### Independent Testing of Model Performance With Test Dataset

**Figure 5:**
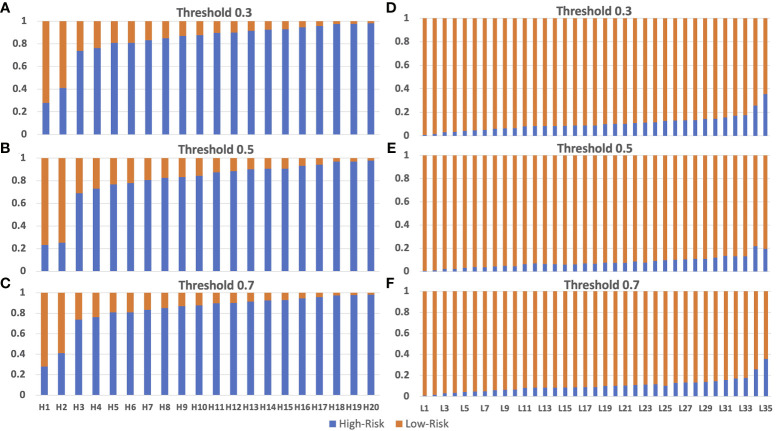
Model testing using the independent testing set of high-risk and low-risk patients. Panel **(A–C)** are from the truely high-risk patients. Panel **(D–F)** are from the truely low-risk patients. Model prediction for high-risk patients with threshold 0.3 **(A)**, 0.5 **(B)** and 0.7 **(C)** had incorrect preditions on patients H1 and H2 which is lower than 0.5. Model prediction for low-risk patients with threshold 0.3 **(D)**, 0.5 **(E)** and 0.7 **(F)** are 100% correct on all low-risk patients. The size of the bar with color corresponding to the correct prediction is the accuracy of the model. Blue color and orange color represent for high-risk and low-risk prediction, respectively.

We validated the model performance with the testing dataset, consisting of 125,401 patches from 55 breast cancer patients (20 patients with high risk and 35 patients with low risk). We used two approaches to evaluate the model prediction performance, namely patch-wise and patient-wise, because in clinical practice, each patient would have 3-5 WSIs for final risk assessment. Both patch-wise and patient-wise methods have high confidence (>85% accuracy), but the patient-wise method is better for clinical use because it provides higher confidence in the model prediction based on the 70-gene signature score in clinical applications. We used only the X_Cept model for this evaluation step, owing to its highest performance in the training and validation phases. The model performance in the independent testing set is reported in **Table 2**. For the patch-wise approach, the precision, recall, and F1 score of the high-risk group were 0.86, 0.85, and 0.85, while these metrics in the low-risk group were 0.89, 0.89, and 0.89, respectively. Both the macro average and the weighted average were 0.87. The patient-wise results for each individual are displayed in **Figures 5A–F**. The model accuracy was consistent across different selected thresholds. A minor shift in the high-risk group was found between the chosen thresholds. Sample H2 with high-risk shifted 16% of prediction probability from 0.25 to 0.41. Another sample from low risk group (L35) also reported a 16% difference of prediction probability from threshold 0.5 relative to 0.3 and 0.7 thresholds. However, the final prediction results for these 2 samples were still unchanged. Overall, the model accuracy was 90% and 100% for the high-risk and low-risk groups, respectively. The overall accuracy reached 96.3% (53/55).Model performance in the independent test set.

**Table 2:** Model performance in the independent test set.

Metrics	Precision	Recall	F1-score	Number of patches
High-risk	0.86	0.85	0.85	54122
Low-risk	0.89	0.89	0.89	71279
Accuracy			0.87	125401
Macro average	0.87	0.87	0.87	125401
Weighted average	0.87	0.87	0.87	125401

### Visualization of Model Prediction

**Figure 6:**
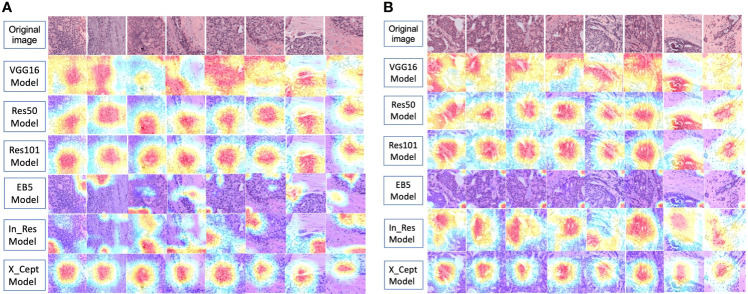
GRAD-CAM visualization of model prediction. **(A)** Visualization of 6 models’ prediction for the same set of images from 8 high-risk patients. **(B)** Visualization of 6 models’ prediction for the same set of images from 8 low-risk patients. The activation level is shown with a gradient of red, yellow, and blue, which represents high, moderate, and low activation of the risk class, respectively.

To decode the model learning process, we used GRAD-CAM with the last activation layer to create a heatmap superimposed on the original image (**Figures 6A, B**). This illustrates how each model learned to distinguish differences between the low-risk and high-risk 70-gene signature scores. VGG16, Res50, Res101, and X_Cept were activated on the tumor part of the patches.

In_Res was activated in the tumor and peri-tumor stroma areas. EB5 was mainly activated on peri-tumor stroma areas. The activated areas of VGG16, Res50, Res101, and X_Cept were highly identical, while the size of the activated area of X_Cept was smaller than those of Res50, Res101, and VGG16. It is readily seen that the VGG16 model’s heatmap wase highly activated over the entire image, whereas the Res50 and Res101 models’ heatmaps were activated in the middle of the image. These three models performed well with the last two images on the right-hand side, which had the majority of cells distributed in the lower and upper corners; however, the models still managed to recognize these areas as tumor cells, which demonstrated the models’ capability and logic in distinguishing tumor and non-tumor areas. The EB5 and In_Res models’ activation maps did not show reasonable pathological features in the images, perhaps because these models used a corner-based approach to determine the difference between low-risk and high-risk groups. Finally, the X_Cept model had a clear activation pattern with clustering of tumor cell areas for both high- and low-risk groups.

## Discussion

Breast cancer recurrence not only negatively affects patients’ quality of life, but a majority of breast cancer patients also undergo chemo- and radiotherapy, which have a high cost and a high rate of side effects (37, 38). Predicting the risk of recurrence for patients diagnosed with breast cancer in an early stage could help in making a suitable treatment plan, which could also prevent overtreatment of patients with highly toxic chemotherapy. In past decades, identification of novel prognostic biomarkers for recurrence was based on multi-omics approaches, which required multi-step protocols and time-consuming analyses (39). Consequently, a rapid, robust, and highly accurate method is highly desirable. Lately, with the emergence of AI tools, machine learning and deep learning have shown promising results in almost every aspect of healthcare research, and they have demonstrated their indispensable roles in assisting and facilitating physicians and researchers in their routine duties.

In an attempt to predict breast cancer recurrence, we have combined deep learning and pathological images into a simple, yet comprehensive and highly accurate, AI pipeline. We developed a complete pipeline for predicting breast cancer recurrence using a single source of data, namely, pathological images with high and low risk scores provided by an established 70-gene signature. Six different pretrained models were used for transfer learning with pretrained weights from the ImageNet dataset. The highest model performance using X_Cept architecture (40) and two fully connected layer of 1024 and 256 neurons achieved an AUC of 0.90 using the validation set and an accuracy of 0.87 using the testing dataset. Furthermore, we bypassed the necessity for region-of-interest/tumor labeling for each WSI, which was a tedious and laborious task for pathologists.

The benefit of the 70-gene signature risk score to breast cancer patients has been proven in several studies with large sample size (11, 13). The important role of this test in assisting physicians with making treatment plans has been affirmed. Nevertheless, mRNA expression profiling of all 70 genes in the signature is needed for completing this test, which is costly and laborious. In addition, discrepancies between different ethnic groups may also lead to divergence in the expression level of certain genes (41, 42), which may affect the risk assessment. Using pathological images as the training data possesses many advantages over gene expression data, such as rapidity, straightforwardness, low cost, and reusability. On top of that, deep learning uses a representation approach to learn from data (43), which has been extensively proven to be superior to human experts in many biomedical tasks such as predicting cancer metastasis from expression data (44), distinguishing diseased and normal cells (45), and detection of tumor areas (46), just to name a few. In our study, we used WSIs for prediction that also contained adjacent tumor areas which may exhibit morphological changes in breast cancers. These adjacent tumor areas contributed to model building because they were different between samples with low- and high-risk 70-gene signature scores. The morphological changes might be small in scale, but deep learning methods, especially their CNN architecture, are designed with hundreds of filters and different kernel sizes, which are particularly practical in detecting these tiny alterations.

The applications of AI tools for biomedical data have been extensively evaluated recently. Hundreds of researchers use different types of data, such as images and omics data, together with clinical information. The performance of these models varies depending on the prediction and classification problem. For instance, in colorectal cancer outcome prediction, a deep learning model achieved an AUC of 0.69, whereas human experts achieved an AUC of 0.58 (30). In another study on non-small cell lung cancer, experts attempted to predict the mutation status of target genes such as TP53 and KRAS using pathological images, and the AUC ranged from 0.733 to 0.856 in the external validation dataset (47). In addition to pathological images, recent studies also used computed tomography scan images for deep learning model training and achieved an AUC of 0.75 for predicting lung cancer treatment response (48). In breast cancer research, various studies have also used deep learning to predict patient outcomes by integrating pathological images and genomic data and have achieved AUCs ranging from 0.681 to 0.821 (49). Interestingly, pathological images were also used to predict the gene expression level in a pan-cancer study, and AUC scores ranged from 0.65 to 0.98 depending on the type and subtype of cancer. In another large-scale study using 44,732 WSIs from 15,187 patients to predict clinical grade from pathological images, a deep learning model achieved a state-of-the-art AUC of 0.98 for all types of cancers. This study had the distinction of using the reported diagnoses for image labeling only (50). The prognostic 70-genes signature achieved 89%, 42%, and 65% for sensitivity, specificity, and overall accuracy, respectively (51, 52). In contrast, our best model (X_Cept model) achieved a sensitivity of 0.89 and a specificity of 0.86 (Based on **Table 2**). Taken together, the feasibility and efficiency of using pathological images and AI tools to predict clinical information and patient outcomes have been demonstrated, and the next step is to conduct prospective studies for evaluating potential application in clinical practice.

With rapid advances in AI algorithms coupled with new hardware generations and a plethora of ready-to-use healthcare data, models can now be trained with larger datasets in a shorter period of time. As a matter of course, expensive genomic, transcriptomic, proteomic, and metabolomic tests for different clinical purposes such as patient outcomes, survival analyses, and cancer subtyping will inevitably receive assistance from faster and better AI tools. Eventually, AI tools are expected to either completely transform traditional healthcare approaches or create a hybrid form. These advances will help physicians make better and faster decisions in treatment planning that requires a personalized medicine approach.

## Conclusion

In the present study, we developed a high-performance, automated deep neural network pipeline to predict risk of breast cancer recurrence using pathological images, which reduces the cost and time of genetic testing and obviates the need for tumor region labeling. We also demonstrated that a deep neural network model could learn the complex pathological features only from images and was able to find tumor areas for distinguishing low- and high-risk breast cancers.

### Limitation

One of the limitations of this study is the size of the dataset and the study populations. Although the models can reach upto 87% accuracy for the patch-wise approach and upto 96.3% for the patient-wise approach, more rigorous independent validations are required to establish their efficacy and and reliability for future applications into bigger datasets from different study groups of varied ethnicities. In addition, the current study has not performed survival analysis, owing to no event in our study cohort.

## Data Availability Statement

The datasets presented in this article are not readily available because the patient’s data are from Taipei Veteran General Hospital and are prohibited from distribution for public use. Requests to access the datasets should be directed to C-CH, chisheng74@gmail.com.

## Ethics Statement

The studies involving human participants were reviewed and approved by Internal review board of the Taipei Veteran General Hospital. The patients/participants provided their written informed consent to participate in this study.

## Author Contributions

Conceptualization: NP, C-YH, and EC. Methodology: NP and C-CH. Validation: NP and C-CH. Formal analysis: NP. Investigation: NP, C-YH, and C-CH. Resources: EC and L-MT. Data curation: NP and C-CH. Writing, original draft preparation: NP, C-YH, and C-CH. Writing, review and editing: NP, CY-H, and C-CH. Visualization: NP, C-YH, and C-CH. Supervision: EC and L-MT. Project administration: EC and L-MT. Funding acquisition: EC and L-MT. All authors have read and agreed to the published version of this paper.

## Funding

This work has been supported in part by the Center of Genomic and Precision Medicine, National Taiwan University, the Ministry of Science and Technology, Taiwan [Grant No. MOST-110-2634-F-002-044], and the Center for Biotechnology, National Taiwan University, Taiwan [grant number GTZ300]. This study was also partly funded by the Taipei Veterans General Hospital grant (V110E-005-3).

## Conflict of Interest

The authors declare that the research was conducted in the absence of any commercial or financial relationships that could be construed as a potential conflict of interest.

## Publisher’s Note

All claims expressed in this article are solely those of the authors and do not necessarily represent those of their affiliated organizations, or those of the publisher, the editors and the reviewers. Any product that may be evaluated in this article, or claim that may be made by its manufacturer, is not guaranteed or endorsed by the publisher.

## Abbreviation

Res50, ResNet50 model; Res101, ResNet101 model; EB5, EfficientNetB5 model; In_Res, Inception_ResnetV2 model; X_Cept, Xception model.

## References

[1] Waks AG Winer EP. Breast Cancer Treatment: A Review. JAMA (2019) 321(3):288–300. doi: 10.1001/jama.2018.19323 30667505

[2] Weigelt B Peterse JL Van't Veer LJ. Breast Cancer Metastasis: Markers and Models. Nat Rev Cancer (2005) 5(8):591–602. doi: 10.1038/nrc1670 16056258

[3] Esteva FJ Hortobagyi GN. Prognostic Molecular Markers in Early Breast Cancer. Breast Cancer Res (2004) 6(3):1–10. doi: 10.1186/bcr777 15084231 PMC400674

[4] Yerushalmi R Woods R Ravdin PM Hayes MM Gelmon KA. Ki67 in Breast Cancer: Prognostic and Predictive Potential. Lancet Oncol (2010) 11(2):174–83. doi: 10.1016/S1470-2045(09)70262-1 20152769

[5] Ivshina AV George J Senko O Mow B Putti TC Smeds J. Genetic Reclassification of Histologic Grade Delineates New Clinical Subtypes of Breast Cancer. Cancer Res (2006) 66(21):10292–301. doi: 10.1158/0008-5472.CAN-05-4414 17079448

[6] Desmedt C Haibe-Kains B Wirapati P Buyse M Larsimont D Bontempi G. Biological Processes Associated With Breast Cancer Clinical Outcome Depend on the Molecular Subtypes. Clin Cancer Res (2008) 14(16):5158–65. doi: 10.1158/1078-0432.CCR-07-4756 18698033

[7] Chia SK Bramwell VH Tu D Shepherd LE Jiang S Vickery T. A 50-Gene Intrinsic Subtype Classifier for Prognosis and Prediction of Benefit From Adjuvant Tamoxifen. Clin Cancer Res (2012) 18(16):4465–72. doi: 10.1158/1078-0432.CCR-12-0286 PMC3743663 22711706

[8] Viale G Slaets L Bogaerts J Rutgers E Van't Veer L Piccart-Gebhart M. High Concordance of Protein (by IHC), Gene (by FISH; HER2 Only), and Microarray Readout (by TargetPrint) of ER, PgR, and HER2: Results From the EORTC 10041/BIG 03-04 MINDACT Trial. Ann Oncol (2014) 25(4):816–23. doi: 10.1093/annonc/mdu026 PMC3969556 24667714

[9] Krijgsman O Roepman P Zwart W Carroll JS Tian S de Snoo FA. A Diagnostic Gene Profile for Molecular Subtyping of Breast Cancer Associated With Treatment Response. Breast Cancer Res Treat (2012) 133(1):37–47. doi: 10.1007/s10549-011-1683-z 21814749

[10] Wuerstlein R Kates R Gluz O Grischke E Schem C Thill M. Strong Impact of MammaPrint and BluePrint on Treatment Decisions in Luminal Early Breast Cancer: Results of the WSG-PRIMe Study. Breast Cancer Res Treat (2019) 175(2):389–99. doi: 10.1007/s10549-018-05075-x PMC6533223 30796651

[11] Cardoso F Piccart-Gebhart M Van’t Veer L Rutgers E. The MINDACT Trial: The First Prospective Clinical Validation of a Genomic Tool. Mol Oncol (2007) 1(3):246–51. doi: 10.1016/j.molonc.2007.10.004 PMC5543876 19383299

[12] Cardoso F Van't Veer L Rutgers E Loi S Mook S Piccart-Gebhart MJ. Clinical Application of the 70-Gene Profile: The MINDACT Trial. J Clin Oncol (2008) 26(5):729–35. doi: 10.1200/JCO.2007.14.3222 18258980

[13] Soliman H Shah V Srkalovic G Mahtani R Levine E Mavromatis B. MammaPrint Guides Treatment Decisions in Breast Cancer: Results of the IMPACt Trial. BMC Cancer (2020) 20(1):81. doi: 10.1186/s12885-020-6534-z 32005181 PMC6995096

[14] Zou J Huss M Abid A Mohammadi P Torkamani A Telenti A. A Primer on Deep Learning in Genomics. Nat Genet (2019) 51(1):12–8. doi: 10.1038/s41588-018-0295-5 PMC11180539 30478442

[15] Alipanahi B Delong A Weirauch MT Frey BJ. Predicting the Sequence Specificities of DNA-And RNA-Binding Proteins by Deep Learning. Nat Biotechnol (2015) 33(8):831–8. doi: 10.1038/nbt.3300 26213851

[16] Angenent-Mari NM Garruss AS Soenksen LR Church G Collins JJ. A Deep Learning Approach to Programmable RNA Switches. Nat Commun (2020) 11(1):1–12. doi: 10.1038/s41467-020-18677-1 33028812 PMC7541447

[17] Ma J Yu MK Fong S Ono K Sage E Demchak B. Using Deep Learning to Model the Hierarchical Structure and Function of a Cell. Nat Methods (2018) 15(4):290. doi: 10.1038/nmeth.4627 29505029 PMC5882547

[18] Goodfellow I Bengio Y Courville A Bengio Y. Deep Learning Vol. 1. Cambridge, MA, USA: MIT press Cambridge (2016).

[19] Norgeot B Glicksberg BS Butte AJ. A Call for Deep-Learning Healthcare. Nat Med (2019) 25(1):14–5. doi: 10.1038/s41591-018-0320-3 30617337

[20] Miotto R Wang F Wang S Jiang X Dudley JT. Deep Learning for Healthcare: Review, Opportunities and Challenges. Briefings Bioinf (2018) 19(6):1236–46. doi: 10.1093/bib/bbx044 PMC6455466 28481991

[21] Park Y Kellis M. Deep Learning for Regulatory Genomics. Nat Biotechnol (2015) 33(8):825–6. doi: 10.1038/nbt.3313 26252139

[22] Guo Y Shang X Li Z. Identification of Cancer Subtypes by Integrating Multiple Types of Transcriptomics Data With Deep Learning in Breast Cancer. Neurocomputing (2019) 324:20–30. doi: 10.1016/j.neucom.2018.03.072

[23] Janowczyk A Madabhushi A. Deep Learning for Digital Pathology Image Analysis: A Comprehensive Tutorial With Selected Use Cases. J Pathol Inf (2016) 7:29. doi: 10.4103/2153-3539.186902 PMC4977982 27563488

[24] McBee MP Awan OA Colucci AT Ghobadi CW Kadom N Kansagra AP. Deep Learning in Radiology. Acad Radiol (2018) 25(11):1472–80. doi: 10.1016/j.acra.2018.02.018 29606338

[25] Yasaka K Akai H Kunimatsu A Kiryu S Abe O. Deep Learning With Convolutional Neural Network in Radiology. Japanese J Radiol (2018) 36(4):257–72. doi: 10.1007/s11604-018-0726-3 29498017

[26] Lakhani P Sundaram B. Deep Learning at Chest Radiography: Automated Classification of Pulmonary Tuberculosis by Using Convolutional Neural Networks. Radiology (2017) 284(2):574–82. doi: 10.1148/radiol.2017162326 28436741

[27] Rizzo R Fiannaca A La Rosa M Urso A. A Deep Learning Approach to Dna Sequence Classification. In International Meeting on Computational Intelligence Methods for Bioinformatics and Biostatistics. Switzerland: Springer, Cham (2015).

[28] Zhou J Troyanskaya OG. Predicting Effects of Noncoding Variants With Deep Learning–Based Sequence Model. Nat Methods (2015) 12(10):931–4. doi: 10.1038/nmeth.3547 PMC4768299 26301843

[29] Khurana S Rawi R Kunji K Chuang G-Y Bensmail H Mall R. DeepSol: A Deep Learning Framework for Sequence-Based Protein Solubility Prediction. Bioinformatics (2018) 34(15):2605–13. doi: 10.1093/bioinformatics/bty166 PMC6355112 29554211

[30] Bychkov D Linder N Turkki R Nordling S Kovanen PE Verrill C. Deep Learning Based Tissue Analysis Predicts Outcome in Colorectal Cancer. Sci Rep (2018) 8(1):1–11. doi: 10.1038/s41598-018-21758-3 29467373 PMC5821847

[31] Liu D Yu J. Otsu Method and K-Means. In 2009 Ninth International Conference on Hybrid Intelligent Systems. Shenyang, China: IEEE (2009).

[32] Macenko M Niethammer M Marron JS Borland D Woosley JT Guan X. A Method for Normalizing Histology Slides for Quantitative Analysis. In 2009 IEEE International Symposium on Biomedical Imaging: From Nano to Macro. Boston, MA, USA: IEEE (2009).

[33] Muñoz-Aguirre M Ntasis VF Rojas S Guigó R. PyHIST: A Histological Image Segmentation Tool. PLoS Comput Biol (2020) 16(10):e1008349. doi: 10.1371/journal.pcbi.1008349 33075075 PMC7647117

[34] Bradski G Kaehler A. Learning OpenCV: Computer Vision With the OpenCV Library. Sebastopol, California, USA: O'Reilly Media, Inc (2008).

[35] Deng J Dong W Socher R Li L-J Li K Fei-Fei L. Imagenet: A Large-Scale Hierarchical Image Database. In 2009 IEEE Conference on Computer Vision and Pattern Recognition. Miami, FL, USA: Ieee (2009).

[36] Selvaraju RR Cogswell M Das A Vedantam R Parikh D Batra D. Grad-Cam: Visual Explanations From Deep Networks via Gradient-Based Localization. In: Proceedings of the IEEE International Conference on Computer Vision. Venice, Italy: IEEE (2017).

[37] Dent R Trudeau M Pritchard KI Hanna WM Kahn HK Sawka CA. Triple-Negative Breast Cancer: Clinical Features and Patterns of Recurrence. Clin Cancer Res (2007) 13(15):4429–34. doi: 10.1158/1078-0432.CCR-06-3045 17671126

[38] Ahmad A. Pathways to Breast Cancer Recurrence. London, United Kingdom: International Scholarly Research Notices (2013). p. 2013.

[39] Sestak I Cuzick J. Markers for the Identification of Late Breast Cancer Recurrence. Breast Cancer Res (2015) 17(1):1–8. doi: 10.1186/s13058-015-0516-0 25848913 PMC4307995

[40] Chollet F. Xception: Deep Learning With Depthwise Separable Convolutions. In: Proceedings of the IEEE Conference on Computer Vision and Pattern Recognition. Honolulu, HI, USA: IEEE (2017).

[41] Bunyavanich S Grant C Vicencio A. Racial/ethnic Variation in Nasal Gene Expression of Transmembrane Serine Protease 2 (TMPRSS2). Jama (2020) 324(15):1567–8. doi: 10.1001/jama.2020.17386 PMC7489401 32910146

[42] Henderson BE Lee NH Seewaldt V Shen H. The Influence of Race and Ethnicity on the Biology of Cancer. Nat Rev Cancer (2012) 12(9):648–53. doi: 10.1038/nrc3341 PMC3677949 22854838

[43] LeCun Y Bengio Y Hinton G. Deep Learning. Nature (2015) 521(7553):436–44. doi: 10.1038/nature14539 26017442

[44] Bejnordi BE Veta M Van Diest PJ Van Ginneken B Karssemeijer N Litjens G. Diagnostic Assessment of Deep Learning Algorithms for Detection of Lymph Node Metastases in Women With Breast Cancer. Jama (2017) 318(22):2199–210. doi: 10.1001/jama.2017.14585 PMC5820737 29234806

[45] Iqbal MS Ahmad I Bin L Khan S Rodrigues JJ. Deep Learning Recognition of Diseased and Normal Cell Representation. Trans Emerg Telecommunications Technol (2020) p:e4017. doi: 10.1002/ett.4017

[46] Shkolyar E Jia X Chang TC Trivedi D Mach KE Meng MQ-H. Augmented Bladder Tumor Detection Using Deep Learning. Eur Urol (2019) 76(6):714–8. doi: 10.1016/j.eururo.2019.08.032 PMC6889816 31537407

[47] Coudray N Ocampo PS Sakellaropoulos T Narula N Snuderl M Fenyö D. Classification and Mutation Prediction From non–Small Cell Lung Cancer Histopathology Images Using Deep Learning. Nat Med (2018) 24(10):1559–67. doi: 10.1038/s41591-018-0177-5 PMC9847512 30224757

[48] Xu Y Hosny A Zeleznik R Parmar C Coroller T Franco I. Deep Learning Predicts Lung Cancer Treatment Response From Serial Medical Imaging. Clin Cancer Res (2019) 25(11):3266–75. doi: 10.1158/1078-0432.CCR-18-2495 PMC6548658 31010833

[49] Sun D Li A Tang B Wang M. Integrating Genomic Data and Pathological Images to Effectively Predict Breast Cancer Clinical Outcome. Comput Methods Programs Biomed (2018) 161:45–53. doi: 10.1016/j.cmpb.2018.04.008 29852967

[50] Campanella G Hanna MG Geneslaw L Miraflor A Silva VWK Busam KJ. Clinical-Grade Computational Pathology Using Weakly Supervised Deep Learning on Whole Slide Images. Nat Med (2019) 25(8):1301–9. doi: 10.1038/s41591-019-0508-1 PMC7418463 31308507

[51] Buyse M Loi S Van't Veer L Viale G Delorenzi M Glas AM. Validation and Clinical Utility of a 70-Gene Prognostic Signature for Women With Node-Negative Breast Cancer. J Natl Cancer Institute (2006) 98(17):1183–92. doi: 10.1093/jnci/djj329 16954471

[52] Tian S Roepman P Van't Veer LJ Bernards R De Snoo F Glas AM. Biological Functions of the Genes in the Mammaprint Breast Cancer Profile Reflect the Hallmarks of Cancer. Biomark Insights (2010) 5:BMI. S6184. doi: 10.4137/BMI.S6184 PMC2999994 21151591

